# Complex Analysis of Antioxidant Activity, Abscisic Acid Level, and Accumulation of Osmotica in Apple and Cherry In Vitro Cultures under Osmotic Stress

**DOI:** 10.3390/ijms22157922

**Published:** 2021-07-25

**Authors:** Petra Jiroutova, Zuzana Kovalikova, Jakub Toman, Dominika Dobrovolna, Rudolf Andrys

**Affiliations:** 1 Research and Breeding Institute of Pomology Ltd., Holovousy 129, 50801 Hořice, Czech Republic; 2 Department of Biology, Faculty of Science, University of Hradec Kralove, Rokitanskeho 62, 50003 Hradec Kralove, Czech Republic; zuzana.kovalikova@uhk.cz (Z.K.); jakub.toman@uhk.cz (J.T.); dominika.dobrovolna@uhk.cz (D.D.); 3 Department of Chemistry, Faculty of Science, University of Hradec Kralove, Rokitanskeho 62, 50003 Hradec Kralove, Czech Republic; rudolf.andrys@uhk.cz

**Keywords:** abiotic stress, in vitro culture, antioxidants, sugar alcohols, proline, abscisic acid

## Abstract

Plant response to osmotic stress is a complex issue and includes a wide range of physiological and biochemical processes. Extensive studies of known cultivars and their reaction to drought or salinity stress are very important for future breeding of new and tolerant cultivars. Our study focused on the antioxidant activity, accumulations of osmotica, and the content of abscisic acid in apple (cv. “Malinové holovouské”, “Fragrance”, “Rubinstep”, “Idared”, “Car Alexander”) and cherry (cv. “Regina”, “Napoleonova”, “Kaštánka”, “Sunburst”, “P-HL-C”) cultivated in vitro on media containing different levels of polyethylene glycol PEG-6000. Our results indicated that the studied genotypes responded differently to osmotic stress manifested as reduction in the leaf relative water content (RWC) and increment in the activities of antioxidant enzymes, proline, sugars, and abscisic acid content. Overall, cherry cultivars showed a smaller decrease in percentage RWC and enzymatic activities, but enhanced proline content compared to the apple plants cultivars. Cultivars “Rubinstep”, “Napoleonova”, and “Kaštánka” exhibited higher antioxidant capacity and accumulation of osmoprotectants like proline and sorbitol that can be associated with the drought-tolerance system.

## 1. Introduction

Plants are constantly exposed to various types of stress, of which the most severe is stress produced by water imbalance in the ecosystem. Both decreasing rainfall and rising average temperatures reduce water storage in soil and cause drought stress. Moreover, irrigation with high ion content water and overuse of chemical fertilizers significantly contributes to soil salinization. Drought and high soil salinity lead to osmotic stress in plants and represent the major abiotic stress factor that affects the growth of plants and agricultural production worldwide [1]. Responses to abiotic stress in plants are complex and include numerous physiological and biochemical processes affecting their growth and productivity. This complexity makes selection and breeding of tolerant cultivars extremely difficult [2].

In general, osmotic stress causes a wide range of morphological, physiological, and biochemical changes that are crucial for mitigating the negative effect of water deficiency. One of the known mechanisms that plants use to avoid water deficit in salt or drought stress environment is osmotic adjustments which are crucial for maintaining cell turgor and plant metabolic activity. Plants synthetize osmotica such as proline or soluble sugars to establish osmotic balance at the cellular level [3]. Proline is a cyclic amino acid with low molecular weight and high water solubility. Under the normal conditions, it is an important part of many proteins and under various stress conditions proline has an essential role as one of the most abundant osmoprotectants. Proline accumulation can be used as a physiological indicator of plant resistance to stress tolerance [4]. Soluble sugars like glucose, fructose, sucrose, and sugars like glycerol or sorbitol represent an essential metabolite complex playing various roles in plant development. They are a fuel for plant growth, signalling molecules, and precursors for metabolism. They can stabilize the cell membrane or protect plats from oxidative stress under the stress conditions by being part of the reactive oxygen species scavenging system [5]. Similar to accumulation of proline, the accumulation of sugars can be used as a physiological indicator for evaluation of stress tolerance [3].

All stress conditions can induce an accumulation of reactive oxygen species (ROS), such as hydrogen peroxide H_2_O_2_ and superoxide ·O_2_^−^, in plant cells. Overproduction of ROS is toxic for plants because they cause the oxidation of lipids, proteins, nucleic acids, and carbohydrates that can result in irreversible changes in plants or lethality. Plants have evolved complex antioxidant system to protect cellular membranes and organelles from the damaging effect of ROS that consists of both enzymatic and non-enzymatic mechanisms of detoxification. Non-enzymatic antioxidant system includes both water soluble low molecular mass compounds, such as ascorbic acid or glutathione, and lipid soluble antioxidants such as α-tocopherol and β-carotene [6]. Enzymatic antioxidant system in plants includes superoxide dismutase (SOD), catalase (CAT), ascorbate peroxidase (APX), and glutathione reductase (GR) [7]. Superoxide dismutase belongs to the first line of enzymatic defence against enhanced level of ROS with its ability to catalyse conversion of superoxide ·O_2_^−^ to O_2_ and H_2_O_2_ in all subcellular compartments. In plants, several distinct types of SOD can occur, each varying with respect to the metal at the active site [8]. Catalase is present in the peroxisomes, and as a key antioxidant enzyme plays a fundamental role in the catalytic scavenging of H_2_O_2_ and its decomposition to water and oxygen. An increase in catalase activity is often part of the adaptive response of plants to abiotic stress. It is assumed that without increase in catalase activity, plant growth would be more reduced and damaged under the stress conditions [9]. Ascorbate peroxidase presents another key antioxidant enzyme involved in scavenging H_2_O_2_ by its conversion to H_2_O using ascorbate as the source of reducing power. The level of expression of APX tightly correlates with the intensity and duration of applied stress. It is suggested that crosstalk amongst various antioxidant enzymes does exist in plants. For example, activity of APX increases in the presence of other antioxidant enzymes like SOD and GR [10].

In addition to antioxidants and osmotica, the phytohormones also remarkably contribute to the adaptation of plants in response to osmotic stress. Among plant hormones, abscisic acid (ABA) plays a central role in the regulation of stress responses via regulation of several physiological processes such as opening and closure of stomata or helping the roots to increase their absorption area. The level of endogenous ABA as well as the ABA associated enzymes sharply increases during water deficit [11,12].

Assessing plant response to osmotic stress can be achieved by using water-soluble, high molecular weight polymer polyethylene glycol (PEG). In the present study, PEG-6000 was added to the cultivation medium. Previously, it has been reported that PEG does not enter the cell wall space and that PEG molecules with a molecular weight greater than 3000 are evidently not absorbed. Apparently, PEG also does not have any toxic effects on plants, and it is widely used to induce drought or salinity stress in higher plants through lowering the water potential of the nutrient solution [13].

Apple is one of the most consumed and widespread agricultural commodities around the world, primarily because of its texture, flavour, and nutritive values [14]. As well as apples, cherries are a popular fruit, valued for both their taste and nutrients with plenty of beneficial health effects [15]. In this study, five apple and cherry cultivars were cultivated at different levels of PEG in medium and their physiological and biochemical responses were monitored. Such a comprehensive analysis of versatile responses to osmotic stress in apple and cherry in vitro culture has not been carried out previously. In addition, this study aims to help breeders find a quick laboratory tool for initial screening of varieties in breeding of new cultivars. Thus, we provide fundamental information for breeding of new resistant or tolerant cultivars. In terms of breeding programs, in vitro based techniques with PEG enriched medium are able to screen diverse plant cultures in laboratory conditions in a short time. On the other hand, the major disadvantage of this method is simulation of drought stress via osmotic stress, contrary to withholding irrigation methods based on whole plants. Therefore, the combination of both methods could be beneficial for future breeding programs.

## 2. Materials and Methods

### 2.1. Plant Material and Experimental Conditions

All the genotypes of apple (*Malus × domestica*) and cherry (*Prunus avium*) were produced in vitro from donor shoots collected in the Research and Breeding Institute of Pomology, Holovousy Ltd., Czech Republic. Cultivars were selected to include a representative of the commercial varieties (“Idared”, “Rubinstep”, “Regina”) but also landraces (“Malinové holovouské”, “Car Alexander”, “Napoleonova”). According to stress tolerance of selected cultivars, apple cultivars “Fragrance”, “Rubinstep”, and “Car Alexander” are considered to be frost resistant [16,17,18] contrary to “Idared”, which is ranked among the sensitive varieties [19]. In case of cherry cultivars, “Sunburst” and “P-HL-C” are resistant to frost [20,21] and “Napoleonova” is known for its drought resistance [22]. In vitro explants were cultivated in 100 mL Erlenmeyer flasks capped with aluminium foil, each flask contained 25 mL of solid (7.0 g L^−1^ agar) Murashige and Skoog (MS) medium [23] with the shoot growth stimulant 6-aminobenzylpurine (BAP) at a concentration of 1.5 mg L^−1^. pH was adjusted to 5.7 before autoclaving at 121 °C for 15 min. The growth conditions were as follows: photoperiod 16 h light/8 h dark and a temperature 22 ± 1 °C. Well established in vitro cultures were transferred in the fresh medium every 30 days. Different levels of osmotic stress were achieved by adding polyethylene glycol (PEG-6000) at a concentration of 0, 5, 10, 25, and 50 g L^−1^ to the basal medium labelled as PEG0, PEG5, PEG10, PEG25, and PEG50, respectively. These concentrations represent water potential of the medium as follow 0.002 MPa, 0.004 MPa, 0.01 MPa, 0.02 MPa. Explants were treated with PEG-6000 for 30 days, which is a standard subcultivation period for this kind of plant material. After 30 days of cultivation on media (with or without PEG-6000), in vitro explants were used as plant material for following analysis. At least three independent measurements were carried out for each cultivar and each concentration.

### 2.2. Determination of Relative Water Content

Six explants from two independent experiments for each cultivar were weighed immediately (FW) after harvesting to determine the relative water content (RWC). Explants were submerged in distilled water for 4 h and then turgid weight (TW) was measured. Afterwards, the explants were dried in oven at 105 °C to the constant weight to obtain their dry weight (DW). Relative water content was calculated using the following formula [24]:

$$\mathrm{RWC}\;\left(\%\right)=\frac{\mathrm{fresh}\;\mathrm{weight}\;\left(\mathrm{FW}\right)-\mathrm{dry}\;\mathrm{weight}\;\left(\mathrm{DW}\right)}{\mathrm{turgid}\;\mathrm{weight}\;\left(\mathrm{TW}\right)-\mathrm{dry}\;\mathrm{weight}\;\left(\mathrm{DW}\right)}\;\times \;100$$

### 2.3. Determination of Osmotic Regulating Compounds

#### 2.3.1. Proline Content

The content of proline was determined via the sulfosalicylic acid/ninhydrin reaction method described by Bates et al. [25]. Fresh leaf material (300 mg) was extracted in 5 mL of 3% sulfosalicylic acid. After centrifugation at 4500 rpm (10 min), 2 mL supernatant were mixed with 2 mL glacial acetic acid and 2 mL acidified ninhydrin reagent and left incubated for 60 min in a boiling water bath. After rapid cooling to 25 °C, 3 mL toluene was added. The absorbance of coloured toluene fractions was read at 520 nm. Proline (Sigma Aldrich, Darmstadt, Germany) was used as a standard.

#### 2.3.2. Simple Sugars and Sugar Alcohols Content

An aliquot of fresh leaves (300 mg) was homogenized in 80% ethanol and heated at 80 °C for 20 min. The homogenate was centrifuged at 4500 rpm for 10 min. The supernatant was collected in a clean tube, the pellet was resuspended in 50% ethanol and reheated at 80 °C for 20 min. After centrifugation, the supernatants were pooled. This step was repeated once more. The content of sugars (glucose, fructose, sucrose) and sugar alcohols (sorbitol, glycerol) in the combined supernatants determined using UHPLC Infinity II 1290 system (Agilent Technologies, Santa Clara, CA, USA) using a 6470 Series Triple Quadrupole mass spectrometer (Agilent Technologies; electrospray ionization, negative polarity) as detector. The method used a BEH Amide column (2.1 mm × 150 mm, 2.6 µm, Waters, Milford, MA, USA). An isocratic elution program was used for chromatographic separation applying mobile phase comprising 20:80% mixture of 0.05% formic acid in water and 8:2 acetonitrile/methanol mixture. The flow rate was 0.4 mL min^−1^. Ion source parameters: gas temperature 150 °C, gas flow 6 L min^−1^, nebulizer 40 psi, sheath gas temperature 300 °C, sheath gas flow 128 L min^−1^, capillary voltage 2500 V, and nozzle voltage 0 V. The sample injection volume was 1 µL. All standards used were purchased from Sigma Aldrich (Darmstadt, Germany).

### 2.4. Determination of Antioxidant Capacity and Antioxidant Enzymes Activity

Antioxidant capacity of plant extracts was assayed using previously reported [26] discoloration of a purple-coloured solution of DPPH (2,2-diphenyl-1-picrylhydrazyl) with some modifications. The mixture of 60 µmol L^−1^ DPPH in methanol (1.5 mL) and the sample extract (30 µL) was left at room temperature in the dark for 30 min and evaluated spectrophotometrically at 517 nm. The percentage of DPPH scavenging effect was calculated using the formula: % Scavenging = [(A_control_ − A_sample_)/A_control_] × 100, where A_control_ is the absorbance of the solution without extract and A_sample_ is the absorption of the solution with extract.

For detection of enzymatic activities, 200 mg of fresh leaves was homogenized in cooled potassium phosphate buffer (50 mmol L^−1^, pH 7.0). The homogenates were centrifuged at 14,000 rpm for 15 min at 4 °C. The SOD activity (EC 1.15.1.1) was determined following the manufacturer instructions of SOD assay kit (Sigma-Aldrich, catalogue number 19160) and expressed as inhibition rate (%). Superoxide anions generated by oxidation of xanthine reduce nitroblue tetrazolium to nitroblue tetrazolium formazan. The presence of SOD reduces the level of superoxide, which also decreases the level of formazan derivative. The activities of CAT (EC 1.11.1.6) and APX (EC 1.11.1.1) were measured at 240 and 290 nm following the decomposition of H_2_O_2_ and oxidation of ascorbic acid, respectively, and expressed as µmol mg^−1^ proteins and nmol mg^−1^ proteins, respectively [27].

Proteins were quantified according to Bradford’s method [28] at 595 nm with bovine serum albumin (Bio-Rad) as a standard.

### 2.5. Determination of Abscisic Acid Content

Fresh plant material (100 mg) was immediately frozen in liquid nitrogen and stored at −80 °C until used. The extraction of abscisic acid (ABA) was carried out as described previously [29]. Briefly, the extraction used precooled 15:4:1 mixture methanol/water/formic acid at −20 °C. The prepared samples were purified using a Sep-Pak^®^ Plus Short tC18 column (Waters, Dublin, Ireland) and a Strata^TM^-X-C 33 µm column (Phenomenex, CA, Torrance, USA) via elution with methanol. The desired fraction was evaporated to dryness and subsequently analysed using UHPLC-MS. Concentration of abscisic acid in all samples was determined using UHPLC Infinity II 1290 system coupled to a 6470 Series Triple Quadrupole mass spectrometer (Agilent Technologies) with negative electrospray ionization (ESI). The method included a Zorbax RRHD Eclipse plus C18 column (2.1 × 50 mm, 1.8 µm, Agilent Technologies), column temperature 40 °C, injection volume 1 µL, the mobile phase flow rate 0.40 mL min^−1^, and a gradient elution program 0 min–10% A, 1.0 min–10% A, 4.0 min–95% A, 5.0 min 94% A, 5.1 min–10% A, and 6.0 min–10% A in B, where A was Milli-Q water and B was acetonitrile. MS conditions were as follow: gas temperature 300 °C, gas flow 6 L min^−1^, nebulizer 40 psi, sheath gas temperature 400 °C, sheath gas flow 12 L min^−1^, capillary voltage 2000 V, and nozzle voltage 1200 V. Two MRM transitions were monitored: 263.1→219.1, 153.1.

### 2.6. Statistical Analysis

Statistical differences among the treatments and cultivars were evaluated using analysis of variance (ANOVA) followed by Tukey test (*p* < 0.05) using Minitab v. 19 software (Minitab LLC, Coventry, UK). Number of replications (n) in tables/figures denotes number of individual samples measured for each parameter.

## 3. Results

**Figure 1:**
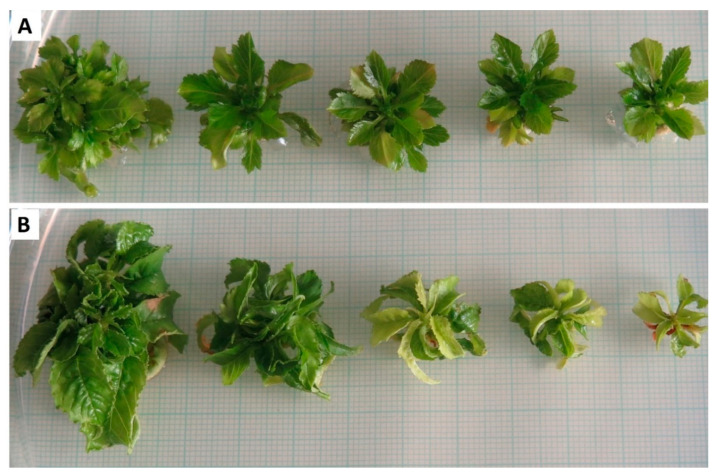
Effect of different concentration of polyethylene glycol (PEG-6000) on the growth and appearance of the in vitro culture of apple cultivar “Rubinstep” (**A**) and cherry cultivar “Kaštánka” (**B**); PEG concentration in g L^−1^ increasing from left to right (PEG0, PEG5, PEG10, PEG25, PEG50).

Generally, the appearance of the in vitro explants changed with the increasing concentration of PEG in culture medium. Rising levels of oxidative stress caused obvious pigment loss and reduction in the explant size, as shown in Figure 1.

### 3.1. Relative Water Content (RWC)

**Figure 2:**
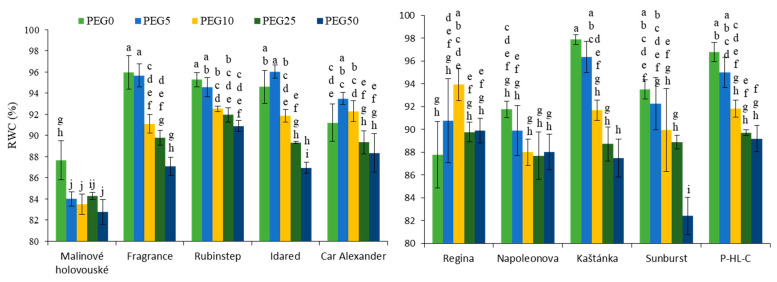
Effect of PEG-6000 concentration on the relative water content (%) for in vitro culture of apple (**left**) and cherry (**right**) cultivars. Error bars represent standard deviation (SD). Values within column, followed by the same letter(s), are not significantly different according to Tukey’s test (*p* < 0.05).

Increasing concentration of PEG in the medium led to a simultaneous decrease in RWC, except for the cultivars “Car Alexander” and “Regina”, where the two lowest concentrations of PEG resulted in a slight enhancement (significantly only in “Regina” PEG10 plants) (Figure 2). Overall, cherry cultivars displayed a decrease to a lower percentage in the tissues RWC than the apple cultivars. The lowest RWC values in control and PEG treated plants of all tested cultivars were monitored in “Malinové holovouské”. A significant decrease compared to untreated plants was observed at higher concentrations of PEG (PEG25 and PEG50), mainly for “Fragrance” and “Idared” apples and “Kaštánka”, “Sunburst” and “P-HL-C” cherries.

### 3.2. Osmotic Regulating Compounds

**Figure 3:**
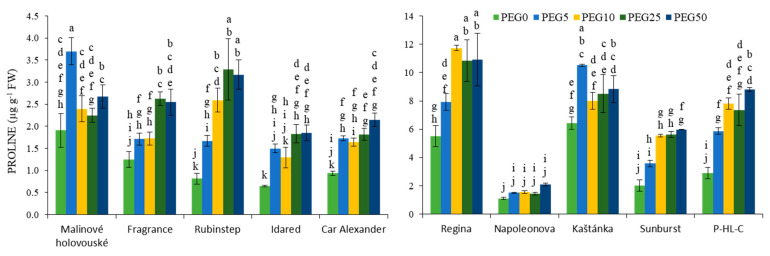
Effect of PEG-6000 concentration on proline content (µg g^−1^ FW) for in vitro culture of apple (**left**) and cherry (**right**) cultivars. Error bars represent standard deviation (SD). Values within column, followed by the same letter(s), are not significantly different according to Tukey’s test (*p* < 0.05).

Increasing concentration of PEG in medium stimulated the proline accumulation in all tested apple and cherry cultivars (Figure 3). The maxima for individual cultivars were achieved mostly after application of the two highest concentrations PEG25 and PEG50, except for “Malinové holovouské” apple cultivar and for “Regina” and “Kaštánka” cherry cultivars. Overall, the measured values were significantly higher for cherries (both control and stressed plants), except for the cultivar “Napoleonova”, where the values were comparable to apple cultivars. A significantly higher increment was monitored in apples, specifically in “Rubinstep” with about 4 times in PEG25 and PEG50 variants. An almost threefold increase was also recorded for “Idared” in the same variants. In the case of cherries, the accumulation of proline was the most pronounced in cultivars “Sunburst” and “P-HL-C” (almost 3-fold for PEG50 plants).

**Figure 4:**
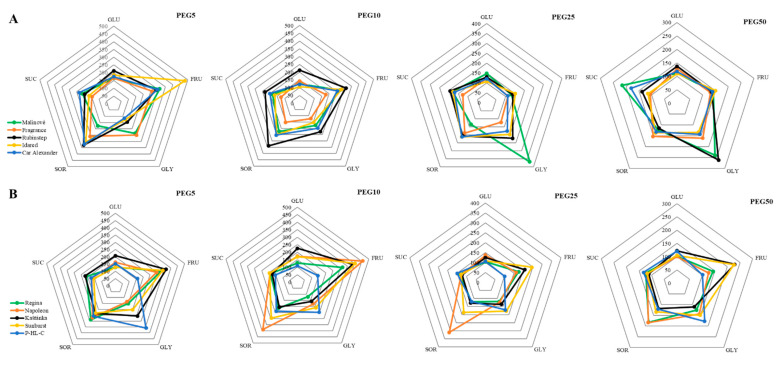
Effect of PEG-6000 concentration on sugars content (mg g^−1^ FW) for in vitro culture of apple (**A**) and cherry (**B**) cultivars. All values were recalculated relative to the compound content in untreated samples taken as 100%. GLU, glucose; FRU, fructose; GLY, glycerol; SOR, sorbitol; SUC, sucrose. The statistical data are shown in Supplementary Material Tables S1 and S2.

In case of the tested apple explants, a sharp increment in monitored sugars content after the application of the two lowest doses of PEG was observed in almost all varieties (Figure 4, detailed data are given in Supplementary Materials Tables S1 and S2). Subsequently, a decrease, sometimes to the control values, was noted. The maximum increase in the content of simple sugars was detected for glucose in cultivars “Rubinstep” and “Kaštánka” (more than 2 times). The maximum fructose contents exhibited apple cultivars “Idared” (almost 5 times), followed by the “Rubinstep” (3 times) and “Napoleonova” (almost 5 times) and “Sunburst” (4 times) cherry cultivars. The highest values of sucrose were recorded for “Rubinstep” and “Car Alexander” apples, and “Kaštánka” and “Regina” cherries.

We followed the changes in the concentration of two sugar alcohols. Glycerol maximum was recorded for apple cultivars only at higher PEG concentrations, specifically in PEG25 for “Malinové holovouské” (3.7 times) and in PEG50 for “Rubinstep” (more than 2.5 times). The maxima in cherries at a PEG5concentration ranged to “P-HL-C” (3.5-fold) and “Kaštánka” with more than 2.5-fold increase. A more than 3-fold increase in second sugar alcohol sorbitol was observed in the varieties “Rubinstep”, “Idared”, and “Napoleonova”. The maxima differed between both individual sugars and varieties. Thus, we could not determine unambiguously in which variety the synthesis of sugars was most stimulated. However, the above results show that the most often mentioned apple cultivar included “Rubinstep”, and cherry cultivars “Napoleonova” or “Kaštánka”.

### 3.3. Antioxidant Capacity and Antioxidant Enzymes Activity

**Figure 5:**
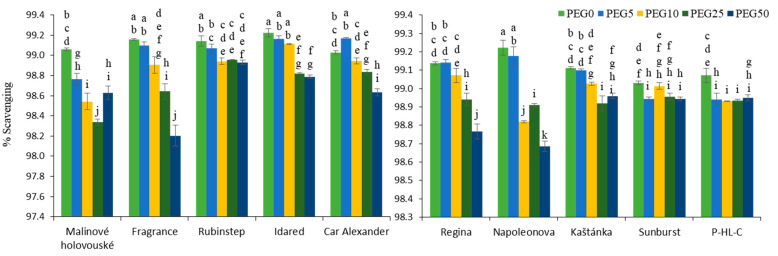
Effect of PEG-6000 concentration on scavenging activity of DPPH radical (%) for in vitro culture of apple (**left**) and cherry (**right**) cultivars. Error bars represent standard deviation (SD). Values within column, followed by the same letter(s), are not significantly different according to Tukey’s test (*p* < 0.05).

Our results show that the plants synthesized a sufficient amount of antioxidants and their total antioxidant capacity, measured as scavenging of DPPH radical, decreased only slightly with the increasing PEG concentration. The most significant differences were noticed in apple cultivars “Fragrance” and “Malinové holovouské” and in cherry cultivars “Regina” and “Napoleonova” (Figure 5).

**Figure 6:**
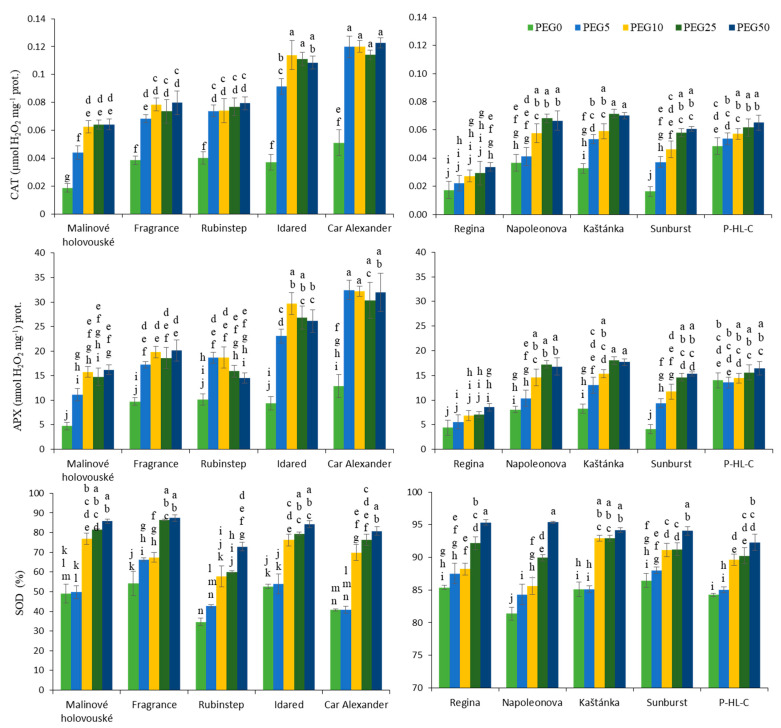
Effect of PEG-6000 concentration on catalase activity CAT (µmol H_2_O_2_ g^−1^ FW), ascorbate peroxidase activity APX (nmol H_2_O_2_ g^−1^ FW), and superoxide dismutase activity SOD (%) for in vitro culture of apple (**left**) and cherry (**right**) cultivars. Error bars represent standard deviation (SD). Values within column, followed by the same letter(s), are not significantly different according to Tukey’s test (*p* < 0.05).

A simultaneous increase in activities of monitored enzymes with increasing PEG concentration was noticed in tested apple and cherry cultivars (Figure 6). The highest values of CAT activity in both PEG0 and PEG treated plants were observed in the cultivar “Car Alexander”. Higher values of enzymatic activity in PEG0 plants were also found in the case of cultivars “Fragrance” and “Rubinstep”. However, at the same time, the smallest increase due to increasing PEG concentration in the medium was observed for them. Overall, CAT activity was mostly stimulated in “Malinové holovouské”, more than 3-fold at PEG50. Catalase activity in cherries was generally lower compared to apples. The highest increase was recorded in the cultivars “Sunburst” (3.7 times) and “Kaštánka” (more than two times), while the lowest increment exhibited “P-HL-C”. Overall, low values of enzymatic activity of untreated and treated plants were recorded in the cultivar “Regina”.

There was a sharp increase in APX activity in apples after application of the lowest PEG dose that further enhanced with increasing PEG concentration. The lowest values were observed again in the cultivar “Malinové holovouské” where the rose was also the highest (up to 3.5 times). APX activity was significantly stimulated in “Idared” as well (up to 3-fold) and “Car Alexander” (2.5-fold). APX activity values within cherry cultivars displayed a similar accumulation trend as in activity of CAT. Overall, the values were lower compared to apples and the increase in activity was gradual. The lowest values of PEG0 and PEG-treated plants were observed in the cultivar “Regina”. The most stimulated enzymatic activity was recorded in “Sunburst” (more than 3.5-fold).

The most significant increment in SOD activity in apple explants was recorded in cultivars “Rubinstep” and “Car Alexander” (approximately 2-fold). At the same time, these varieties had the lowest enzymatic activity seen for PEG0 plants. On the contrary, the highest values in PEG0 plants were typical of cultivars “Fragrance” and “Idared” and the lowest increment in activity was recorded due to an increase in concentration of PEG in the medium. The increment in SOD activity was less pronounced in cherries. The highest values were recorded for the “Napoleonova”, but only a 1.2-fold increase. At the same time, the lowest activity of PEG0 plants was observed in this case. Overall, it can be noted that SOD activity, both with and without the addition of PEG, was significantly lower in cherries.

### 3.4. Abscisic Acid Content

**Figure 7:**
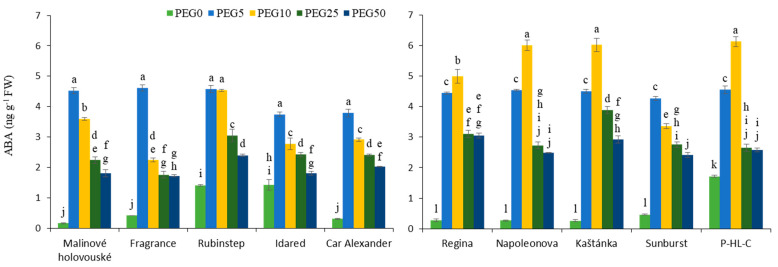
Effect of PEG-6000 concentration on abscisic acid content ABA (ng g^−1^ FW) for in vitro culture of apple (**left**) and cherry (**right**) cultivars. Error bars represent standard deviation (SD). Values within column, followed by the same letter(s), are not significantly different according to Tukey’s test (*p* < 0.05).

The addition of PEG in the medium was associated with a significant increase in ABA content, compared to control (PEG0). The increase of PEG concentration over 5% for apple and over 10% for cherry explants caused a significant reduction of ABA content. The highest increase, up to 27 times, was observed for apple cultivar “Malinové holovouské”. For cherry cultivars “Napoleonova” and “Kaštánka” more than 20-fold rose was monitored. Overall, higher levels in PEG0 plants were in the “Rubinstep”, “Idared”, and “P-HL-C”, while simultaneously the lowest increase in ABA content of only 2.5–3.5-fold after the PEG application was observed (Figure 7).

## 4. Discussion

Apples and cherries are among the most cultivated fruit trees both in the Czech Republic and Europe. The total yield of these trees is affected by water availability. The growing number of dry days during the vegetative season and worldwide increasing shortages of water led to the search for drought-resistant cultivars. The response of plants to limited water supply are monitored at multiple levels [30]. In the present study, we studied biochemical responses of apple and cherry in vitro cultures to induced drought-like stress conditions.

Leaf water potential and RWC are usually used as important and reliable indices of plant water deficit or water status. In agreement with our finding, various studies reported a significant decline in RWC in several fruit species including apple rootstock [31], pistachio trees [32], almond [33,34], and *Prunus* rootstock [35], as well as in cherry rootstock, and fig in vitro cultures [36,37]. Moreover, the authors suggested that water potential in resistant genotypes under water deficit condition is maintained longer than susceptible genotypes. For example, Turkan et al. [38] pointed out that the RWC level in the leaves of resistant cultivars were not affected by drought stress, while in sensitive cultivars, the RWC was reduced up to 10% in 14 days. The drought sensibility out of our tested cultivars was declared for “Malinové holovouské” and “Car Alexander” [17,39] that, however, was not manifested in a significant decrease in RWC. In contrast, the decline in RWC was relatively small in the tolerant cherry cultivar “Napoleonova” [22].

The influence of abiotic and biotic stress stimuli, including water deficit, leads to an imbalance between ROS production and degradation. Their elevated accumulation affects plant metabolism in different ways and leads to cellular damage. The antioxidant machinery in plants is a result of both sequential and simultaneous actions of various antioxidant molecules together with antioxidant enzymes including SOD, CAT, and APX [6,10]. Our experiments confirmed that activities of these enzymes were elevated in drought-stressed apple and cherry leaves compared with the controls. The peak activities were monitored at the two highest PEG concentrations. Similar results were also found for five kiwifruit species [40], apple trees [31], and sweet cherry in vitro explants [36], where the more pronounced answer was in drought tolerant genotypes. Here, on the other hand, the activities of APX and other enzymes involved in ascorbate-glutathione cycle reacted markedly differently. The drop under mild and severe stress conditions could result from the elevated ROS production and its interaction with the above-mentioned enzymes that lead to its inactivation via quick oxidation. These enzyme activations and the magnitude of it appears decisive in the development of stress sensitivity and tolerance. For example, the activation of CAT and APX is stronger in tolerant species, while in contrast, sensitive species increasingly activate GPX [38,41].

In our results, it is not possible to state quite clearly which varieties are more tolerant due to changes in the accumulation of the above enzymes. Due to the sharp increase in the activities of CAT, APX, and SOD, “Malinové holovouské” and “Car Alexander” appeared as more tolerant apple cultivars. However, these findings contradict the declared low or very low drought resistance, respectively [17,39]. The cultivars “Fragrance” and “Rubinstep” with declared frost resistance, which may also be related to increased drought resistance, showed an overall low increase in CAT and APX activities due to increasing PEG concentration in medium. However, overall, the values in both PEG0 and PEG treated plants were high compared to other cultivars, and thus the ROS quenching potential was high, which could be reflected in a low increase in enzymatic activities. On the contrary, in cherry, tolerant cultivar “Napoleonova” [22] corresponded to the above proposed concept of increased enzymatic activity due to water stress conditions. At the same time, the very frost-resistant “Sunburst” [20] showed a similar accumulation trend.

It is well known that plants accumulate wide scale of osmoregulators such as inorganic ions, soluble sugars, proline or glycine betaine in the vacuole and cytosol that contribute to membrane stability thus reducing the negative effect of water stress [3]. We followed the accumulation changes of two main representatives, proline and sugars. In our present study, proline content significantly increased with increasing PEG concentration in the medium. Other authors also reported elevated proline accumulation in various plants under in vitro conditions [33,34,36] and field experiments [35]. However, it is important to note here that Kautz et al. [42] pointed out that proline values could vary significantly with respect to the method of cultivation and the induction of drought-like conditions. Proline concentration in the hydroponically grown seedlings did not match the values those for plants cultivated in soil and was more than two times higher.

In addition to its osmoprotective role, proline also acted as a ROS scavenger and molecular chaperone contributing to stabilization of protein structure and protection of the cells from damage caused by adverse environments [43]. Therefore, it was suggested that genotypes with higher proline content may be more resistant to stressful conditions, as was reported in different pistachio [32] and grass pea [44] genotypes. If we leaned towards this statement, cultivars “Malinové holovouské”, “Fragrance”, “Regina” and “Kaštánka” would appear to be more tolerant. However, the rate of biosynthesis and the activity of individual genes could also play a role in the rapid accumulation of proline and thus increased resistance. For example, gene P5SC, codifying crucial enzyme in biosynthesis, was also up-regulated and its higher expression was correlated with higher proline levels in drought-tolerant *Prunus* and safflower cultivars in comparison with a drought-sensitive one [35,45]. Thus, genetic manipulations of proline synthesis and degradation leading to its increased levels in plants might be a promising tool for enhanced drought resistance in plants [46].

Carbohydrates, besides their important function as energy rich molecules and building block for plant growth, play a crucial role in processes maintaining cell turgor under osmotic stress, e.g., under drought and salinity. Changes in the concentrations of water-soluble carbohydrates, mostly sugars such as glucose, fructose, sucrose, sorbitol, and mannitol, can be a result of the regulation of sugar metabolism and export [47,48]. Levels of these sugars were affected by different types of drought-induced stress. A concentration-dependent experiment with pistachio demonstrated a simultaneous increase in total soluble sugars and sucrose with increasing PEG concentration in the medium where tolerant cultivars reacted more markedly [32]. Only the content of sorbitol in apples, but not of other soluble carbohydrates, responded to drought in the same way as in our study, i.e., a significant increase at moderate stress. However, its concentration varied under severe drought and was less or similar to that in controls [49]. Time dependent water studies significantly stimulated the synthesis of sorbitol, glucose, fructose, and galactose in apple trees [47] and *Prunus* rootstock [35]. On the contrary, sucrose concentration was significantly lower in the drought-stressed variants. Given the high concentration of leaf sorbitol, authors suggested that sorbitol rather than sucrose is preferentially accumulated at the low photosynthetic rate of drought-stressed plants and acts as a storage of carbon. The accumulation behaviour of sucrose makes it more available sugar than sorbitol. These results were also supported by both the expression and activity of key enzymes aldose-6-phosphate reductase (A6PR) [47] and sorbitol-6-phosphate dehydrogenase (S6PDH) [35] in sorbitol synthesis. Moreover, sucrose acts as a signal molecule participating in the crosstalk between hormonal, oxidative, and defence signalling [50].

Owing to the putative role of proline and sorbitol as antioxidants, they could be ameliorating deleterious effects of drought-induced oxidative stress by protecting membranes and enzymes. These osmoprotectants, together with higher activity of antioxidant enzymes, show these genotypes as drought resistant [51,52].

Abscisic acid is regarded as a stress hormone as a result of its rapid accumulation under unfavourable environmental conditions. Therefore, overall higher ABA concentrations in plants can be associated with improved stress tolerance that helps the plant to survive [53]. In the present study, the endogenous ABA content in the leaves of apple and cherry explants rose with drought treatment and reached maximum when the two lowest PEG concentrations were used. Similar results were found for poplar trees where three different watering regimes significantly increased ABA content [41]. Time dependent studies showed that prolonged drought conditions resulted in gradual increase in ABA content [54,55]. In addition, the expression of genes related to the ABA biosynthesis pathway correlated with physiological results of drought response, i.e., with a decrease in photosynthesis activity as consequence of ABA-induced stomatal closure. The expression was higher after 15 days under drought conditions [56]. Other processes on a metabolic level, catabolism, or de/conjugation, also controlled the level of stress induced ABA accumulation as proven by elevated levels of phaseic acid, dihydrophaseic acid, and ABA-glucose ester [55,57].

Potentially drought-sensitive cultivars of apple [58] and pepper [59] reacted more readily. The rootstock itself can also have an influence on the overall formation of drought tolerance. Tworkoski et al. [57] and Zhang et al. [60] pointed out that different cultivars drafts on a more tolerant rootstock accumulated more ABA. This type of cultivation, together with the production of plans with enhanced expression of ABA genes, can lead to the development of more drought resistant varieties. According to this statement, “Malinové holovouské”, “Fragrance”, “Rubinstep” in apples, and “Napoleonova”, “Kaštánka” and “P-HL-C” in cherries accumulated the highest amounts of ABA. Besides the first mentioned, all cultivars are drought or frost tolerant.

## 5. Conclusions

Our work focused on the relative water content, antioxidant activity, accumulations of osmotica, and the content of abscisic acid in apple and cherry cultivated in vitro on media containing increasing levels of polyethylene glycol and causing different levels of osmotic stress in tested plants. Our results confirmed that studied genotypes responded differently to osmotic stress manifested as reduction in the leaf relative water content and increment in the activities of antioxidant enzymes, as well as contents of proline, sugars, and abscisic acid. Generally, cherry cultivars exhibited a lower decrease in RWC and enzymatic activities but a higher proline content than the apple plants. Based on our findings, we concluded that apple cultivar “Rubinstep” and cherry cultivars “Napoleonova” and “Kaštánka” could be evaluated as drought tolerant since they exhibited the major features including higher antioxidant capacity and accumulation of osmoprotectants that are crucial for plant stress-tolerance. To our best knowledge, such a comprehensive analysis of mentioned responses to osmotic stress in apple and cherry in vitro culture, as we produced, has never been published. It provides fundamental information for breeders. Our study also suggests a fast method for prediction of the plant tolerance under controlled laboratory conditions that can be used for the initial screening of fruit cultivars.

## Data Availability

The data presented in the current study are available in the article and supplementary materials.

## Supplementary Materials

The following are available online at https://www.mdpi.com/article/10.3390/ijms22157922/s1, Table S1. Effect of different concentration of PEG 6000 on sugars of in vitro culture of five apple cultivars. Data are means ± SDs. Values within column, followed by the same letter(s), are not significantly different according to Tukey’s test (*p* < 0.05). Table S2. Effect of different concentration of PEG 6000 on sugars of in vitro culture of five cherry cultivars. Data are means ± SDs. Values within column, followed by the same letter(s), are not significantly different according to Tukey’s test (*p* < 0.05).
