# Supplementary material for: Complex Analysis of Antioxidant Activity, Abscisic Acid Level, and Accumulation of Osmotica in Apple and Cherry In Vitro Cultures under Osmotic Stress
Source: Int J Mol Sci. 2021 Jul 25;22(15):7922. doi: 10.3390/ijms22157922 (PMC8347229; doi:10.3390/ijms22157922)
Supplement: Supplementary file 1 [file ijms-22-07922-s001.zip › ijms-1261605-SI.pdf]

Table S1. Effect of different concentration of PEG 6000 on sugars of in vitro culture of five apple cultivars. Data are means ± SDs. Values within column, followed by the same letter(s), are not significantly different according to Tukey's test ($p < 0.05$).

Cultivar	PEG Concentration (g·L^{-1})	Glucose (mg·g^{-1} FW)	Fructose (mg·g^{-1} FW)	Glycerol (mg·g^{-1} FW)	Sorbitol (mg·g^{-1} FW)	Sucrose (mg·g^{-1} FW)
Malinové Holovouské	0	1.28 ± 0.04 i	0.66 ± 0.03 p	0.024 ± 0.003 ef	1.24 ± 0.08 j	1.23 ± 0.10 ijkl
	5	2.10 ± 0.19 de	2.04 ± 0.10 fg	0.057 ± 0.005 bc	2.18 ± 0.26 f	2.63 ± 0.02 b
	10	1.60 ± 0.04 gh	1.79 ± 0.01 hi	0.043 ± 0.010 cd	2.78 ± 0.04 e	2.24 ± 0.07 cd
	25	1.88 ± 0.08 ef	0.87 ± 0.04 mno	0.090 ± 0.009 a	1.67 ± 0.04 ghi	2.24 ± 0.10 cd
	50	1.42 ± 0.05 hi	0.93 ± 0.02 lmn	0.058 ± 0.004 bc	1.51 ± 0.06 hij	2.65 ± 0.04 b
Fragrance	0	1.27±0.07 i	0.83 ± 0.01 nop	0.027 ± 0.001 ef	1.20 ± 0.09 j	1.36 ± 0.04 ij
	5	2.11±0.05 de	2.18 ± 0.03 fg	0.067 ± 0.011 b	3.12 ± 0.06 de	2.01 ± 0.07 def
	10	1.82±0.08 fg	1.49 ± 0.05 j	0.033 ± 0.000 def	1.83 ± 0.01 gh	1.72 ± 0.03 g
	25	1.57±0.05 gh	0.94 ± 0.04 lmn	0.033 ± 0.000 def	2.27 ± 0.03 f	1.73 ± 0.04 g
	50	1.63 ± 0.04 fgh	1.08 ± 0.03 l	0.043 ± 0.002 cd	1.84 ± 0.04 gh	1.44 ± 0.03 hi
Rubinstep	0	1.64 ± 0.03 fgh	1.28 ± 0.04 k	0.025 ± 0.002 ef	1.41 ± 0.03 ij	1.28 ± 0.08 ijk
	5	3.48 ± 0.08 a	3.68 ± 0.10 b	0.037 ± 0.001 def	4.64 ± 0.33 ab	2.51 ± 0.04 b
	10	1.82 ± 0.08 a	4.02 ± 0.05 a	0.057 ± 0.006 bc	4.78 ± 0.09 a	2.98 ± 0.18 a
	25	1.57 ± 0.02 de	1.77 ± 0.01 hi	0.056 ± 0.001 bc	2.91 ± 0.11 de	2.48 ± 0.07 bc
	50	1.63 ± 0.07 d	1.73 ± 0.04 i	0.066 ± 0.008 b	1.62 ± 0.06 ghi	1.75 ± 0.02 g
Idared	0	1.50 ± 0.14 hi	0.68 ± 0.02 op	0.028 ± 0.004 def	1.44 ± 0.08 ij	1.01 ± 0.08 l
	5	2.77 ± 0.15 c	3.29 ± 0.10 c	0.035 ± 0.005 def	4.39 ± 0.05 b	1.69 ± 0.14 gh
	10	1.58 ± 0.03 gh	1.93 ± 0.09 gh	0.043 ± 0.002 cd	3.61 ± 0.10 c	1.69 ± 0.10 g
	25	1.61 ± 0.02 gh	1.03 ± 0.06 lm	0.056 ± 0.004 bc	3.07 ± 0.06 de	1.80 ± 0.07 fg
	50	1.65 ± 0.02 fgh	1.02 ± 0.05 lm	0.038 ± 0.007 de	1.96 ± 0.04 fg	1.15 ± 0.07 jkl
CAR Alexander	0	1.82 ± 0.08 fg	0.93 ± 0.02 lmn	0.022 ± 0.002 f	1.47 ± 0.14 ij	1.04 ± 0.07 kl
	5	3.17 ± 0.03 b	2.77 ± 0.06 d	0.025 ± 0.001 ef	4.72 ± 0.09 ab	2.42 ± 0.07 bc
	10	2.23 ± 0.03 d	2.38 ± 0.07 e	0.043 ± 0.004 cd	3.72 ± 0.05 c	2.09 ± 0.06 de
	25	2.21 ± 0.10 d	1.04 ± 0.05 lm	0.038 ± 0.004 de	3.13 ± 0.08 d	1.78 ± 0.08 fg
	50	2.14 ± 0.05 d	1.29 ± 0.07 k	0.031 ± 0.001 def	1.94 ± 0.05 fg	1.87 ± 0.05 efg

Table S2. Effect of different concentration of PEG 6000 on sugars of *in vitro* culture of five cherry cultivars. Data are means ± SDs. Values within column, followed by the same letter(s), are not significantly different according to Tukey's test ($P < 0.05$).

Cultivar	PEG Concentration ($g \cdot L^{-1}$)	Glucose ($mg \cdot g^{-1}$ FW)	Fructose ($mg \cdot g^{-1}$ FW)	Glycerol ($mg \cdot g^{-1}$ FW)	Sorbitol ($mg \cdot g^{-1}$ FW)	Sucrose ($mg \cdot g^{-1}$ FW)
Regina	0	3.53 ± 0.07 efg	1.28 ± 0.02 l	0.030 ± 0.001 fghijk	1.98 ± 0.05 jkl	1.98 ± 0.10 jkl
	5	4.52 ± 0.10 d	4.22 ± 0.16 bc	0.045 ± 0.005 cd	5.71 ± 0.14 a	4.18 ± 0.05 b
	10	4.52 ± 0.02 d	4.06 ± 0.15 bcd	0.036 ± 0.003 defgh	4.22 ± 0.08 de	3.72 ± 0.09 cd
	25	3.66 ± 0.01 ef	2.24 ± 0.09 ijk	0.036 ± 0.001 defghi	2.37 ± 0.11 i	2.67 ± 0.08 f
	50	3.58 ± 0.07 ef	1.84 ± 0.10 jk	0.038 ± 0.004 cdefg	3.60 ± 0.10 fg	2.44 ± 0.09 fghi
Napoleonova	0	3.44 ± 0.09 fgh	1.10 ± 0.09 l	0.023 ± 0.002 kl	1.21 ± 0.08 m	2.08 ± 0.07 jkl
	5	5.56 ± 0.12 bc	3.28 ± 0.06 fg	0.031 ± 0.002 fghijk	3.26 ± 0.02 gh	3.55 ± 0.05 d
	10	5.96 ± 0.19 ab	5.04 ± 0.19 a	0.041 ± 0.001 cde	4.70 ± 0.08 bc	3.98 ± 0.10 bc
	25	4.71 ± 0.05 d	1.79 ± 0.05 k	0.028 ± 0.001 hijk	3.75 ± 0.18 f	2.65 ± 0.12 f
	50	3.46 ± 0.09 fgh	1.39 ± 0.20 l	0.033 ± 0.002 efghij	2.22 ± 0.25 ij	2.44 ± 0.09 fghi
Kaštánka	0	2.53 ± 0.17 l	1.04 ± 0.04 l	0.026 ± 0.006 jk	1.87 ± 0.06 jkl	2.06 ± 0.14 jkl
	5	5.23 ± 0.21 c	3.80 ± 0.01 de	0.067 ± 0.007 a	4.39 ± 0.08 cde	4.51 ± 0.19 a
	10	5.69 ± 0.28 ab	4.01 ± 0.11 cd	0.041 ± 0.002 cde	3.81 ± 0.14 f	3.60 ± 0.11 d
	25	3.16 ± 0.10 ghi	2.16 ± 0.05 ijk	0.035 ± 0.003 hijk	2.38 ± 0.11 i	2.54 ± 0.17 fgh
	50	3.08 ± 0.10 hij	2.38 ± 0.14 hi	0.029 ± 0.001 efghij	2.21 ± 0.09 ij	2.30 ± 0.11 ghij
Sunburst	0	3.58 ± 0.07 efg	1.09 ± 0.08 l	0.022 ± 0.004 kl	1.65 ± 0.06 l	1.90 ± 0.07 l
	5	4.60 ± 0.25 d	3.72 ± 0.04 de	0.045 ± 0.004 cd	3.83 ± 0.06 f	3.01 ± 0.09 e
	10	6.08 ± 0.09 a	4.39 ± 0.17 b	0.047 ± 0.003 bc	4.88 ± 0.20 b	3.71 ± 0.07 cd
	25	3.90 ± 0.19 e	2.68 ± 0.09 h	0.039 ± 0.002 cdef	3.07 ± 0.10 h	2.45 ± 0.02 fghi
	50	3.74 ± 0.03 ef	2.46 ± 0.09 hi	0.033 ± 0.005 efghij	2.21 ± 0.10 ij	2.24 ± 0.08 hijk
P-HL-C	0	2.47 ± 0.08 l	2.20 ± 0.05 ij	0.016 ± 0.000 l	1.77 ± 0.11 kl	1.47 ± 0.19 m
	5	3.76 ± 0.10 ef	3.53 ± 0.18 ef	0.056 ± 0.002 b	4.58 ± 0.09 bcd	2.59 ± 0.01 fg
	10	2.69 ± 0.13 jkl	3.15 ± 0.25 g	0.039 ± 0.002 cdef	4.22 ± 0.11 e	2.27 ± 0.04 ghij
	25	2.62 ± 0.04 kl	2.23 ± 0.09 i	0.026 ± 0.001 ijk	2.10 ± 0.07 ijk	2.19 ± 0.13 ijkl
	50	2.97 ± 0.09 ijk	2.24 ± 0.08 i	0.028 ± 0.002 hijk	2.14 ± 0.09 ij	1.93 ± 0.08 kl
